# The dark side of browning

**DOI:** 10.1007/s13238-017-0434-2

**Published:** 2017-07-04

**Authors:** Kirstin A. Tamucci, Maria Namwanje, Lihong Fan, Li Qiang

**Affiliations:** 1 Institute of Human Nutrition, College of Physicians and Surgeons, Columbia University, New York, NY 10032 USA; 2 Department of Pathology and Cell Biology, Naomi Berrie Diabetes Center, College of Physicians and Surgeons, Columbia University, New York, NY 10032 USA

**Keywords:** adipocyte, browning, beige adipocyte, thermogenesis, obesity, diabetes

## Abstract

The induction of brown-like adipocyte development in white adipose tissue (WAT) confers numerous metabolic benefits by decreasing adiposity and increasing energy expenditure. Therefore, WAT browning has gained considerable attention for its potential to reverse obesity and its associated co-morbidities. However, this perspective has been tainted by recent studies identifying the detrimental effects of inducing WAT browning. This review aims to highlight the adverse outcomes of both overactive and underactive browning activity, the harmful side effects of browning agents, as well as the molecular brake-switch system that has been proposed to regulate this process. Developing novel strategies that both sustain the metabolic improvements of WAT browning and attenuate the related adverse side effects is therefore essential for unlocking the therapeutic potential of browning agents in the treatment of metabolic diseases.

## Introduction

Adipose tissue is sensitive to changes in nutrient supply and ambient temperature: an evolutionary development that has allowed animal species to adapt to food shortage and cold temperatures. In higher vertebrates, white adipose tissue (WAT) primarily stores energy in the form of triglycerides in unilocular white adipocytes, which can then be released as fatty acids when food is scarce (Zechner et al. 2012). In this way, endothermic animals are able to sustain their energy homeostasis long enough to survive through nutritional privation and maintain their core body temperature (Gesta et al. 2007; Zechner et al. 2012). On the other hand, brown adipose tissue (BAT) dissipates energy as heat in a process called non-shivering thermogenesis (Cannon and Nedergaard 2004). Brown adipocytes contain multilocular lipid droplets, densely packed mitochondria, and have a high expression of uncoupling protein 1 (UCP1). BAT is therefore highly metabolically active due to the uncoupling of electron transport from ATP production in the inner mitochondrial membrane, allowing for active substrate oxidation and a low rate of ATP production with heat generation instead.

BAT was previously known to be abundant only in hibernating mammals, interscapular regions of rodents, and supraclavicular regions of human newborns (SMITH and Hock 1963; Aherne and Hull 1966; Rothwell and Stock 1979). However, this has been displaced by the discovery of active BAT in the axillary, cervical, supraclavicular, and paravertebral regions of adult humans (Nedergaard et al. 2007; Cypess et al. 2009; van Marken Lichtenbelt et al. 2009; Virtanen et al. 2009). Together, brown and white adipose tissues orchestrate energy balance and thermal regulation in endothermic animals.

## Beige adipocytes and browning

The accumulation of ‘brown-like’ adipocytes in WAT is referred to as ‘browning’ or ‘beiging’. These ‘brown-like’ adipocytes are referred to as beige or brite (brown-in-white) adipocytes, the activation of which upregulates Ucp1 and other genes involved in energy expenditure in WAT. Browning of WAT is an adaptive and reversible response to environmental stimuli, including cold exposure, pharmacological agents such as β_3_-adrenergic receptor agonists and thiazolidinediones (TZDs), as well as various peptides and hormones (Guerra et al. 1998; Himms-Hagen et al. 2000; Barbatelli et al. 2010; Fisher et al. 2012; Ohno et al. 2012; Rosenwald et al. 2013). Interestingly, characterization of BAT from adult humans has been shown to have a molecular profile more similar to beige fat than that of classical BAT (Wu et al. 2012; Frontini et al. 2013; Sidossis et al. 2015).

Beige adipocytes have multiple origins. They can originate from progenitors resident within WAT that are differentiated in response to browning stimuli—a process known as *de novo* differentiation (Wang et al. 2013). These beige adipocyte progenitors are smooth muscle-like pericytes that express platelet-derived growth factor (PDGF) receptor α but not Myf-5 (PDGFRα^+^; Myf*5* ^−^) (Seale et al. 2008; Lee et al. 2012; Sanchez-Gurmaches et al. 2012; Long et al. 2014). Alternatively, beige adipocytes can arise via transdifferentiation, a process that involves the direct conversion of existing white adipocytes into brown-like cells, and vice versa (Barbatelli et al. 2010; Rosenwald et al. 2013). In sum, beige adipocytes possess distinct phenotypic and functional characteristics from white and brown adipocytes that are underlain by their unique gene expression signature in response to environmental stimuli.

## The regulation of browning

### Transcriptional regulation

Cellular energy sensing and sympathetic tone are the driving forces that regulate the transcriptional networks controlling browning. Peroxisome proliferator-activated receptor γ (PPARγ) centers the browning transcriptional network. It has been proven to be necessary and sufficient for adipocyte differentiation and function (Farmer 2006). Chronic stimulation of primary adipocyte cultures with thiazolidinediones (TZD), a class of PPARγ ligands, induces activation of the PPARγ cofactor, PGC-1α (Wilson-Fritch et al. 2004), and stabilizes the BAT-specific cofactor, PR domain zinc finger protein 16 (PRDM16) (Ohno et al. 2012). In mice, Prdm16 stimulates the expression of several genes involved in thermogenesis in WAT, including *Pgc*-*1α* and *Ucp1*, even after stimulation by β_3_-adrenergic agonists (Seale et al. 2007; Seale et al. 2008). Vernochet et al. further demonstrated a direct role for PPARγ in the phenotypic conversion of WAT to BAT. Specifically, a mutation of the PPARγ ligand-binding domain suppressed TZD-mediated inhibition of white-adipocyte genes, including *Resistin*, *Angiotensinogen* and *Chemerin*, and induced brown-specific genes, including *Ucp-1*, in 3T3-L1 adipocytes (Vernochet et al. 2009). Such inhibition depends on the expression of C/EBPα and the corepressors, carboxy-terminal binding proteins 1 and 2 (CtBP1/2). On the molecular level, TZDs induce deacetylation of PPARγ by the NAD-dependent protein deacetylase sirtuin-1 (SirT1) to recruit browning cofactors such as PRDM16. This results in the selective activation of brown genes and the repression of white genes (Qiang et al. 2012).

Modulations of PPARγ through ligands, posttranslational modifications, isoform distinction (Li et al. 2016), and cofactor exchanges are all able to regulate browning. For example, EBF2 (Rajakumari et al. 2013) and TLE3 (Villanueva et al. 2011) were recently identified as brown and white adipocyte-specific regulators, respectively. Both of them function through PPARγ (Villanueva et al. 2013; Ferrannini et al. 2016). Another browning factor, IRF4, induces thermogenic activity in WAT by activating PRDM16 and PGC-1α, both of which are closely related to PPARγ (Kong et al. 2014). Taken together, PPARγ coupled with its upstream and downstream regulators comprises the browning regulatory axis.

### Hormonal regulation

Cold exposure and other environmental stimuli elicit complex hormonal responses that facilitate adaptive thermogenesis and crosstalk between tissues. For example, lipid-derived hormones—such as prostaglandins, bone morphogenetic protein 4 (BMP-4), and fibroblast growth factor 21 (FGF21)—are produced in response to β_3_-adrenergic receptor activation to promote browning (Vegiopoulos et al. 2010; Fisher et al. 2012; Grefhorst et al. 2015). Furthermore, leptin is a nutrient-responsive adipokine that, together with insulin, promotes browning through POMC neurons (Dodd et al. 2015). The browning effect of leptin is counteracted by another representative adipokine: Adiponectin (encoded by *Adipoq*). *Adipoq* knock-out mice show increased thermogenic response (Qiao et al. 2014), in line with the decreased energy expenditure in its transgenic mice on ob/ob background (Kim et al. 2007). In addition, catecholamines are required for the immediate activation of brown and existing beige adipocytes, as well as for the differentiation of beige adipocytes from their precursors (Cannon and Nedergaard 2004). Adipose-tissue resident M2 macrophages were identified as a source of catecholamines involved in the regulation of lipolysis in response to acute cold exposure (Nguyen et al. 2011). Obesity induces a switch toward proinflammatory M1 macrophages (Lumeng et al. 2007) that might counteract the increased catecholamine production and therefore prevent browning. However, the production of catecholamines by adipose M2 macrophages was questioned in a recent study (Fischer et al. 2017), suggesting a revisit to the immune-regulation of browning. Moreover, secretory factors are suggested to mediate the crosstalk between muscle and fat in terms of exercise-induced brown remodeling in WAT (Moghri et al. 2013). Overall, it is agreed that energy sensing and metabolic demands are important regulators of the browning process. Various environmental stimuli cause the release of hormonal factors from adipose tissue and/or other metabolically active organs, all of which contribute to the maintenance of energy homeostasis.

Besides the mainstreams of transcriptional and hormonal regulations, various mechanisms have been identified to regulate browning that include cytoskeleton remodeling (McDonald et al. 2015), circadian rhythm (Gerhart-Hines et al. 2013), microRNAs (Kim et al. 2014), long non-coding RNAs (Alvarez-Dominguez et al. 2015), and the central nervous system (Liu et al. 2012; Hankir et al. 2016). Despite the continuously growing list of browning regulators, our opinion is that the challenge is not in identifying new browning factors but rather in understanding the precise mechanisms of browning in order to translate them safely and efficiently into clinical applications.

## The metabolic benefits of browning

### Browning in humans

Obesity confers an increased risk of developing insulin resistance, type 2 diabetes mellitus, and cardiovascular disease (Van Gaal et al. 2006; Guilherme et al. 2008). Browning of WAT has a number of positive implications for metabolic health by tilting the energy balance toward energy expenditure. Thus, stimulation of the activity of brown and beige adipocytes has gained considerable attention for its therapeutic potential in promoting overall metabolic health as recorded by reduced body weight, adiposity, insulin resistance, and hyperlipidemia. In humans, BAT mass, or indeed beige fat mass, and its functional activity are inversely related to body mass index, resting plasma glucose, and lipid levels (Saito et al. 2009). Conversely, increasing BAT activity by cold exposure, diet, or pharmacological agents is positively correlated with energy expenditure. For example, individuals subjected to 10-day cold exposure demonstrated enhanced glucose uptake in BAT, glucose oxidation, and insulin sensitivity (Chondronikola et al. 2014). In a study by Cypess et al., treatment with mirabegron, a β_3_-adrenergic receptor agonist, led to higher BAT metabolic activity and increased basal metabolic rate in healthy male subjects (Cypess et al. 2015). In addition, a 5–8 h exposure of overweight/obese men to a non-shivering cold environment (19.9 ± 0.8°C) activated BAT and increased the expression of lipid handling genes (Chondronikola et al. 2016). These studies suggest a role of beige fat in lipid metabolism, thermogenesis, and energy dissipation. However, it is too early to conclude the therapeutic consequences of inducing browning in humans for the prevention and management of metabolic diseases that include obesity, diabetes, and cardiovascular disease.

### Genetic models of browning

Studies in genetic mouse models have further corroborated the metabolic benefits of browning. Adipose-specific overexpression of *Ucp1* in agouti viable yellow (A^vy^) genetically obese mice resulted in reductions in total body weight and subcutaneous fat stores (Kopecky et al. 1995). These results were supported by another study where mice overexpressing *Ucp1* in adipose tissue were resistant to diet-induced obesity (Stefl et al. 1998). This was attributed to ectopic expression of *Ucp1* in white fat, thus increasing its thermogenic capacity. However, brown fat mass and its *Ucp1* expression were drastically reduced in these mice, indicating that the resistance to obesity was largely due to the increased adaptive thermogenesis in WAT but not in BAT (Stefl et al. 1998). Mice overexpressing *Prdm16* in fat tissue had marked increases of browning in WAT, specifically subcutaneous depots, resulting in protection from diet-induced obesity and glucose intolerance (Seale et al. 2011). Supportively, ablation of Prdm16 in fat impaired browning and led to obesity and insulin resistance (Cohen et al. 2014). Furthermore, overexpression of *Cyclooxygenase 2* (*Cox2*), an enzyme involved in prostaglandin synthesis, also induced browning of WAT and consequently increased energy expenditure and reduced adiposity (Vegiopoulos et al. 2010).

Genes that have been highlighted in cancer, such as *Foxc2*, *Pten*, and *Folliculin*, also have been implicated in browning pathways. Overexpression of *Foxc2* in HFD-fed mice resulted in reduced fat mass as well as protection from the associated insulin resistance and intramuscular accumulation of lipids (Cederberg et al. 2001; Kim et al. 2005). Overexpression of tumor suppressor *Pten* leads to increased energy expenditure, hyperactive BAT, and higher levels of Ucp1 in mice (Ortega-Molina et al. 2012). Folliculin (FLCN) is known for its role as a tumor suppressor and also has been implicated in metabolic reprogramming of adipose tissue (Wada et al. 2016). Ablation of *Flcn* in adipocytes results in increased energy expenditure and protection from diet-induced obesity. This is due to activation of *Ucp1* and other BAT genes in both BAT and WAT, conferring an increased cold tolerance (Yan et al. 2016). Despite these metabolic benefits exhibited by the aforementioned mouse models with induced WAT browning, it remains to be determined whether they confer benefits in terms of eliminating risk to cancer.

## The side effects of browning agents

While the metabolic benefits of browning in humans remain to be fully established, safety is a concern that must first be addressed regarding any method used to induce browning. Cold exposure is a classic and efficient way to induce browning, but its obvious discomfort, together with risks of hypothermia, makes it impractical for clinical use. Therefore, browning agents, either endogenous or exogenous, provide an attractive alternative for improving metabolic diseases. Here we discuss a few commonly used browning agents to draw attention to the safety concern of inducing WAT browning.

### Thiazolidinediones (TZDs)

Thiazolidinediones (TZDs) are PPARγ agonists that were widely used as insulin sensitizers in the treatment of type 2 diabetes. In addition to their insulin sensitizing function, TZDs are well known to induce thermogenic gene expression in both white and brown adipocytes (Sell et al. 2004; Rong et al. 2007; Petrovic et al. 2010; Qiang et al. 2012). Although TZDs have been proven to be effective in the treatment of type 2 diabetes, their use has been limited by the incidence of adverse side effects, some of which include heart failure, edema, weight gain, and bone loss (Shah and Mudaliar 2010; Abbas et al. 2012; Soccio et al. 2014). In this regard, the first clinically available TZD, troglitazone, was withdrawn in 2000, three years after its approval by FDA, due to serious hepatotoxicity (Knowler et al. 2005). Similarly, rosiglitazone was banned in various countries in 2010 due to the increased incidence of heart attack and stroke. Nevertheless, the IRIS (Insulin Resistance Intervention after Stroke) clinical study recently reported a positive outcome in the use of pioglitazone for the treatment of heart disease associated with insulin resistance (Kernan et al. 2016). Non-diabetic patients with insulin resistance along with a recent history of ischemic stroke or transient ischemic attack (TIA) were treated with either pioglitazone or placebo. Pioglitazone was effective in reducing the risk of diabetes by 52% in addition to decreasing the risk of stroke or myocardial infarction by 24%. Despite these promising results, the adverse side effects of TZDs, such as bone loss, weight gain, and edema, were confirmed by this study. This highlights the need to further investigate the mechanisms of TZD action in order to harness the full therapeutic potential of these drugs for insulin sensitization and browning activation.

### FGF21

Fibroblast growth factor 21 (FGF21) emerges as an insulin-mimetic hormone that regulates systemic energy balance and has beneficial effects on body weight, insulin sensitivity, dyslipidemia, and pancreatic β-cell growth (Kharitonenkov et al. 2005; Wente et al. 2006; Kharitonenkov et al. 2007; Coskun et al. 2008; Gaich et al. 2013). Interestingly, FGF21-treated mice show a marked increase in the expression of the key thermogenic genes *Ucp1* and *Dio2* in inguinal WAT (iWAT), whereas *Fgf21*-deficient mice show an impaired response to cold stress due to diminished thermogenic activity (Fisher et al. 2012). The browning capacity of FGF21 is mediated through stabilization of Pgc-1α (Chau et al. 2010; Fisher et al. 2012) or a positive-feedback on PPARγ activation (Dutchak et al. 2012).

One significant limitation to the use of FGF21 as a browning agent is the occurrence of severe bone loss. Wei et al. demonstrated that genetic *Fgf21* gain-of-function, as well as pharmacological FGF21 treatment, in diet-induced obese mice reduced the number and area of osteoblasts and osteoclasts while increasing that of bone marrow adipocytes (Wei et al. 2012). In addition, chronic exposure to FGF21 has been linked to growth retardation in mice based on the development of growth hormone (GH) resistance in *Fgf21*-transgenic mice (Inagaki et al. 2008). Overexpression of *Fgf21* has also been shown to cause infertility in female but not in male mice (Inagaki et al. 2007). Moreover, FGF21 reduces physical activity and promotes torpor in *Fgf21* transgenic mice: a favorable adaptive response to starvation, but an undesirable outcome in the context of obesity (Inagaki et al. 2007). Hence, despite the beneficial effects of FGF21 in terms of improving insulin resistance and inducing browning, its severe side effects will have to be overcome for long-term clinical administration.

### β_3_-Adrenergic receptor agonists

β_3_-adrenergic receptors (β_3_-AR) mediate thermogenesis in BAT and lipolysis in WAT; thus, activating these receptors with selective pharmacological agonists is an attractive strategy for stimulating the browning of WAT. A number of β_3_-AR agonists have been developed as anti-obesity agents. However, their harmful side effects have called into question whether the long-term stimulation of β_3_-ARs is safe and beneficial. Himms-Hagen et al. demonstrated that chronic treatment with a β_3_-AR agonist, CL 316,243, led to the appearance of multilocular brown adipocytes in WAT, promoted thermogenesis, and delayed the development of obesity in rats fed a high-fat diet (Himms-Hagen et al. 1994). However, its browning effects in humans are subtle with chronic administration (Weyer et al. 1998; Arch 2002). Another agonist, Mirabegron, a prescribed drug for treating overactive bladder, has been shown to activate BAT in young, lean, and healthy male humans at a dose of 200 mg/kg/day, but it also causes tachycardia (Cypess et al. 2015). A lower dose appeared safe, as reported by the BEAT-HF trial (Beta 3 Agonists Treatment in Heart Failure), after eliminating the intolerance to adverse events seen at the higher dose (Bundgaard et al. 2016). Therefore, more specific β_3_-AR agonists are desired for the treatment of obesity and diabetes, but their chronic effects must be closely monitored.

### Thyroid hormone

Thyroid hormones (THs) T_4_ (thyroxine) and T_3_ (triiodothyronine) are key regulators of metabolism and energy homeostasis, and have been shown to induce WAT browning (Mullur et al. 2014). Low doses of the T_3_ metabolite, triiodothyracetic acid (TRIAC), induced ectopic expression of UCP1 in rat abdominal WAT (Medina-Gomez et al. 2008). Consistently, chronic administration of GC-1, a thyroid hormone receptor β-specific agonist, to obese mice markedly increased browning of subcutaneous WAT with a significant increase in core body temperature and whole body energy expenditure (Lin et al. 2015). Similar safety concerns for the use of β_3-_AR agonists have been raised for THs in terms of heart risks, hyperthermia, and weight loss (Moolman 2002; Mullur et al. 2014). THs have also been linked to an increased risk of fractures in postmenopausal women with lower serum thyroid-stimulating hormone (TSH) levels (Bauer et al. 2001), which directly affects bone turnover (Murphy and Williams 2004). This further emphasizes the need for browning agents to be carefully designed and controlled in order to ensure its safe metabolic benefits.

### BMP7

Bone morphogenetic proteins-7 (BMP-7) is a member of the superfamily of transforming growth factor-β. It has been shown to singularly promote the differentiation of mesenchymal progenitor C3H10T1/2 cells to a brown adipocyte lineage (Tseng et al. 2008). Treatment of C57Bl6/J mice with BMP7 resulted in the extensive browning of WAT, as evidenced by increased expression of the BAT marker *Ucp1* and the appearance of brown adipocyte clusters (Boon et al. 2013). Most notably, BMP7 treatment of diet-induced obese mice at subthermoneutrality also led to an improved metabolic profile in these mice as demonstrated by reduced fat mass, lower plasma glucose, and hepatic triglycerides (Boon et al. 2013). These results are promising in terms of a potential therapeutic approach for the treatment of obesity. However, it should be noted that BMP7 is approved by the FDA only for clinical practice in long bone trauma, spinal fusion, and oral and maxillofacial applications due to concerns of cancer and immunosuppression (Buijs et al. 2007; Boon et al. 2011; Carreira et al. 2014).

### VEGF-A

VEGF-A is the master angiogenic factor and has been demonstrated to regulate the expansion and homeostasis of fat tissue (Sun et al. 2012; Elias et al. 2012; Lu et al. 2012; Sung et al. 2013). Using an inducible adipocyte-specific VEGF-A overexpression model, Sun et al. demonstrated that the local up-regulation of VEGF-A in adipocytes improved vascularization and led to the browning of WAT, with massive up-regulation of UCP1 and PGC-1α (Sun et al. 2012). This was accompanied by an increase in energy expenditure and resistance to high fat diet-mediated metabolic dysfunction (Sun et al. 2012). On the contrary, loss of VEGF-A in adipose tissue elicits browning of WAT (Lu et al. 2012). However, the consequences of the proangiogenic activity of VEGF-A may not always be beneficial. During adipose tissue expansion, VEGF-A evidently serves a protective role for the metabolically challenged adipose tissue by facilitating browning. In contrast, under conditions of preexisting adipose tissue dysfunction, the stimulation of angiogenesis and fat pad expansion would likely have the opposite—and therefore detrimental effect. For instance, anti-VEGF-A therapies have been applied to treat cancer and eye diseases (Ferrara and Adamis 2016). Thus, the nature of its proangiogenic properties and the related tumorigenic potential impedes the utilization of VEGF-A as a therapeutic browning agent in obesity and diabetes treatment.

## Browning and hypermetabolism

### Cachexia

Cachexia is a metabolic wasting syndrome characterized by severe weight loss, systemic inflammation, and atrophy of WAT and skeletal muscle. It is most commonly observed in cancer patients but has also been associated with burn injuries, infectious diseases (HIV, Tuberculosis), and chronic diseases (congestive heart failure, chronic kidney diseases, chronic obstructive lung disease) (Argilés et al. 2014). Cachexia contributes to the poor prognostic outcomes for these patients and specifically contributes to 20% of cancer-related deaths (Fearon et al. 2013; Argilés et al. 2014). Worse still is that an increase in calorie intake does not improve the cachectic state in patients (Pedroso et al. 2012).

Browning of WAT has primarily been discussed in light of its metabolic benefits: namely, increased energy expenditure, improved insulin sensitivity, and weight loss. However, recent studies have identified browning of WAT as a potential contributor to the development and progression of hypermetabolism in cachexia (Petruzzelli et al. 2014; Kir et al. 2014; Randall et al. 2015; Kir et al. 2016). In the K5-SOS mouse model of skin tumors that exhibits a rapid development of cachexia, energy expenditure was elevated while the respiratory exchange ratios (RER) were reduced, suggesting that lipids were used as the primary energy source in these cachectic mice. This explains the observed increase in catabolism of fatty acids in order to meet the high energy demand in cachexia (SMITH and Hock 1963). On the other hand, attenuation of lipolysis via genetic ablation of adipose triglyceride lipase (ATGL) not only preserves WAT but also prevents muscle wasting (Das et al. 2011). Therefore, browning of WAT in pathologic conditions, such as cancer and burn injury, adds fuel to an already highly catabolic state, leading to a number of deleterious consequences.

### Cachexia factors and browning

#### Interleukin-6

Cachexia has also been described as a highly inflammatory state. There is evidence to suggest that cytokines and potentially other tumor-secreted factors may be responsible for inducing the hypermetabolic state and the consequent reduction in body weight and fat mass (Fearon et al. 2012). Recently, IL-6 has been shown to induce and sustain WAT browning in cachexia. Mice injected with IL-6 proficient C26 carcinoma cells rapidly lost body weight and became cachectic (Petruzzelli et al. 2014). Conversely, blocking IL-6 with a neutralizing monoclonal antibody or with sulindac, a nonsteroidal anti-inflammatory drug (NSAID), reduces the severity of cachexia and suppresses the browning capacity of subcutaneous WAT (Petruzzelli et al. 2014).

#### PTH and PTHrP

Parathyroid-hormone (PTH) and Parathyroid-hormone-related protein (PTHrP) have been implicated in the browning of WAT in cachexia. PTHrP was originally recognized for its beneficial effects on skin, cartilage, placenta, and bone development (Maioli et al. 2002; Guntur et al. 2015). However, its function has recently been associated with hypermetabolic conditions and subsequent detrimental outcomes. Using Lewis lung carcinoma (LLC) cells as a model of cancer cachexia, tumor-derived PTHrP was shown to contribute to wasting by inducing the expression of thermogenic genes, including *Ucp1*, *Dio2*, and *Pgc-1α* (Kir et al. 2014). Treatment of the tumor-bearing mice with a PTHrP-neutralizing antibody inhibited adipose tissue browning and prevented loss of muscle mass and strength. In addition, parathyroid hormone (PTH) was shown to stimulate a thermogenic gene program in 5/6 nephrectomized mice (a model of chronic kidney disease) that suffer from cachexia (Kir et al. 2016). Consequently, fat-selective knockout of its signaling receptor, *PthR*, blocked adipose tissue browning and wasting, preserved muscle mass, and improved strength. In fact, these *PthR* knockout mice were resistant to tumor-induced cachexia (Kir et al. 2016).

### Burn injury

Burn trauma causes hypermetabolism due to marked increases in catecholamines, which have been reported years after the initial injury (Kulp et al. 2010). This sustained increase in catecholamines leads to chronic activation of the β-adrenergic signaling pathway, which in turn initiates browning of WAT and the cascade of events leading to the hypermetabolic response (Sidossis et al. 2015; Patsouris et al. 2015). Most notably, it was recently shown that palmitate, an abundant free fatty acid found in the sera of burn patients, could regulate macrophage polarization (Xiu et al. 2015; Xiu et al. 2016). This interaction may suggest a detrimental feed-forward loop, where browning-induced lipolysis causes free fatty acid efflux, which in turn sustains the browning response during the hypermetabolic state. Further investigation is needed to clarify the function of browning in burn injury, whether it is beneficial to the recovery or contributes to the complications of burn injury. Additionally, a prolonged hypermetabolic state can result in hepatic steatosis and immune suppression (Jeschke 2009; Jeschke et al. 2014). In this regard, the long-term benefits of browning might be canceled out.

## The role of browning in aging

Aging is arguably a major risk factor for metabolic syndrome (Tchkonia et al. 2010) and is accompanied by a loss of active BAT and beige adipocytes in WAT (Cypess et al. 2009; Saito et al. 2009; Rogers et al. 2012). In theory, this loss of browning capacity leads to the reduction in energy expenditure and the expansion of adiposity (Yoneshiro et al. 2011; Rogers et al. 2012), and thus contributes to the progressive metabolic decline associated with aging (Barzilai et al. 2012). The decrease of browning is likely caused by changes in gonadal hormones, desensitization to β-adrenergic signaling (Nedergaard and Cannon 2010) or other factors. Recently, Foxa3 has been identified as a novel transcriptional regulator that inhibits browning during aging (Ma et al. 2014). Although the loss of *Foxa3* resulted in a lean, energy inefficient and more insulin sensitive phenotype in mice older than one year old (Ma et al. 2014), it has been suggested that Foxa3 is a “hoarder” gene to facilitate lipid storage in aged animals (Ma et al. 2015). Indeed, energy preservation is probably more important for survival during food deprivation, which is apparently a challenge for aged animals when their predatory ability declines. Therefore, the metabolic benefits of browning may be unveiled predominately under the conditions of nutrient excess.

## The brake-switch system of browning

In recently years, significant progress has been made in identifying stimuli and signaling pathways that can induce browning of WAT and trigger adaptive thermogenesis. These advancements in knowledge have garnered great support in exploiting adipose tissue plasticity together with browning agents as therapeutic tools for obesity, albeit with side effects as discussed above. The revelation of the two sides of the coin regarding the browning of WAT—namely, that it mitigates the metabolic consequences of obesity but propagates a hypermetabolic state in other pathologic conditions—suggests that browning “wastes energy” and thus is not a favorable physiological state. Therefore, we hypothesized that the body needs to tightly regulate this browning process via a “brake-switch” system to prevent the negative outcomes of both hyper- and hypo-activation of browning activity (Ferrannini et al. 2016).

This “brake-switch” hypothesis is supported by the recent identification of HOXC10, a homeobox domain-containing transcription factor, as a negative regulator of browning of WAT (Ferrannini et al. 2016; Lim et al. 2016). It is enriched in subcutaneous fat, and the ectopic overexpression of HOXC10 suppressed brown fat genes and induced white adipocyte-specific genes with a minimal effect on the pan-adipocyte markers (Ferrannini et al. 2016; Ng et al. 2017). The HOXC10 browning-inhibitory effect is partially mediated by suppressing *Prmd16* gene expression (Ng et al. 2017). Another molecular brake for browning is ZFP423 (Shao et al. 2016), which is a C2H2 zinc-finger protein that had previously been identified as a transcriptional regulator of preadipocyte determination (Gupta et al. 2010). Ablation of *Zfp423* in white adipocytes led to the accumulation of beige adipocytes in WAT in adult mice, while its gain-of-function converted brown adipocytes into a more white-like phenotype (Shao et al. 2016). Taken together, HOXC10 and ZFP423 represent the native “brake” system in white adipocytes to release the browning activity only under appropriate conditions. This switch is likely dysregulated in the hypermetabolic state, as seen in cachexia, or the hypometabolic state, as seen in aging.

## Conclusion

**Figure 1:**
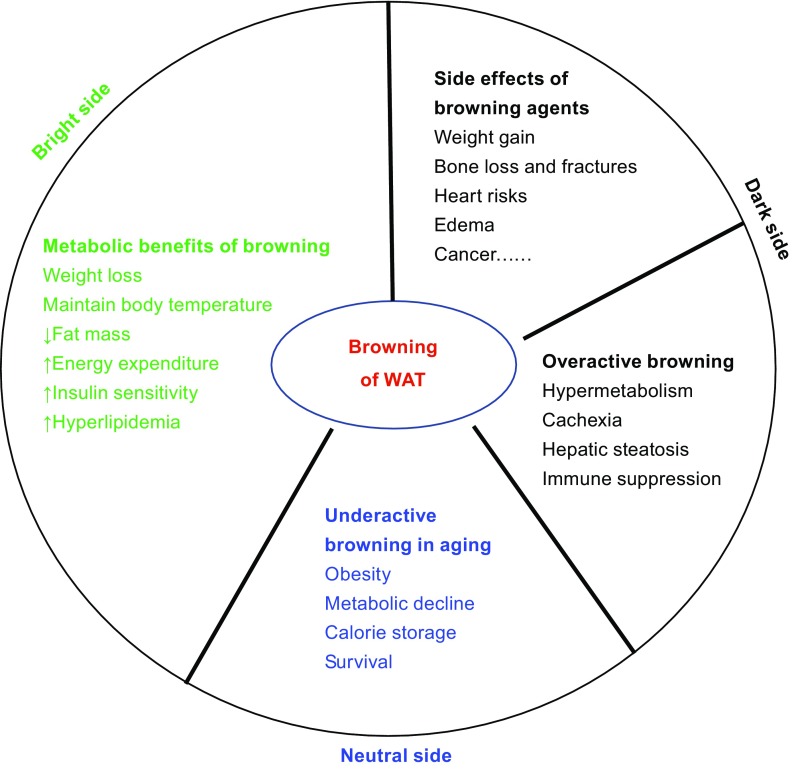
A summary of the metabolic benefits and adverse outcomes associated with the induction of browning

The browning of WAT has become an increasingly favorable strategy for ameliorating the effects of obesity and subsequent metabolic dysfunction. However, it is energetically inefficient and thus is physiologically unfavorable. Furthermore, recent evidence has implicated browning in the development of cachexia, lipotoxicity, and other detrimental outcomes under acute and chronic hypermetabolic conditions (as summarized in Fig. 1). The increasing awareness of the dark side of browning emphasizes the need to further investigate factors and mechanisms that regulate the activation and deactivation of browning. In this regard, the brake-switch system of browning may be critical for maintaining the proper function of BAT and WAT. Further investigation should be warranted to efficiently induce browning in a tissue-specific and tightly controlled manner in order to minimize the occurrence of negative effects and to maximize the therapeutic potential of browning agents in the treatment of metabolic disorders.
